# Barriers and Facilitators to Cognitive Participation in Peer Support for Complementary Feeding in LMICs: A Theory‐Informed Systematic Review

**DOI:** 10.1111/mcn.70154

**Published:** 2026-01-08

**Authors:** Asnake Ararsa Irenso, Hirbo Shore, Karen Campbell, Rachel Laws

**Affiliations:** ^1^ Institute for Physical Activity and Nutrition Deakin University Melbourne Australia; ^2^ Ambo University Ambo Ethiopia; ^3^ Department of Nutrition, Dietetics and Food Monash University Melbourne Australia; ^4^ University of Southern Queensland Toowoomba Australia; ^5^ Institute for Physical Activity and Nutrition, School of Exercise and Nutrition Sciences Deakin University Geelong Australia

**Keywords:** cognition, complementary feeding, developing countries, peer support, volunteers

## Abstract

Suboptimal complementary feeding practices remain a significant challenge in LMICs. Peer support shows promise in improving these practices; however, their long‐term success hinges on sustained engagement and integration into existing support systems, aspects that remain poorly understood. This theory‐led systematic review aimed to understand why people participate, support, and continue using peer support for complementary feeding practices. The literature search covered studies conducted between January 1990 and February 2025. This pragmatic, Normalisation Process Theory‐led review employs an integrative mixed‐methods synthesis. We conducted a theory‐informed systematic review guided by the four subconstructs of cognitive participation, presenting the findings narratively into barriers and facilitators. While peer‐led complementary feeding promotion involves multiple actors at different levels, few studies directly included family members and caregivers beyond mothers, thereby limiting their cognitive participation. Most studies were donor‐driven. Financial, structural, sociocultural, training, and capacity‐related factors strongly influence peer‐led complementary feeding support. Their influences were not fixed; what served as facilitators in the initial stages of studies became barriers later, and vice versa. Household and local leaders and gatekeepers constrained peer support early in the interventions, but this later reversed with their involvement. Incentives boosted interest and increased enrolment. However, this also led to volunteers being less motivated and to attrition when resources were limited. Weak supportive supervision diminished the legitimacy of peer support, causing a loss of confidence in volunteers' skills. Keeping the momentum of early implementation stages requires a predictable funding model, primarily from domestic sources, and sustained engagement in the intervention. This can address multifaceted operational problems, ranging from recruitment to embedding the intervention in the health system. Political commitment, especially when translated into operational support, can strengthen the financial sustainability of peer support programmes.

## Summary

- The sustainability of peer support programmes remains at risk due to over‐dependence on donor funding, necessitating a shift towards domestic resources.
- Incentives boosted interest and increased enrollment, but their practicality under tight resource constraints requires re‐evaluation to develop strategies to prevent volunteer demotivation and attrition.
- Ensuring sustained peer support requires adequate oversight to maintain commitment and early intervention gains; otherwise, strengths previously leveraged can regress into barriers.
- Sustaining the trust and legitimacy built through credible women and existing social networks requires ensuring ongoing skill enhancement for peer support workers.
- Peer support works well within the multimodal complementary feeding strategies.

## 1 Introduction

Suboptimal complementary feeding practices continue to be a global public health concern (Lutter et al. 2021). A review by Kupka et al. (2020) based on data from low‐ and middle‐income countries (LMICs) revealed striking gaps. One‐third of children aged 6–23 months did not start solid foods by 6 months; only 53% met the minimum meal frequency (MMF), and 29% met the minimum dietary diversity (MDD). Only one in five children met both the MMF and the MDD. Dietary quality is lowest for children from families with low incomes, who often struggle to afford it (Kupka et al. 2020).

Social norms influence food choices by guiding actions and behaviours that reflect community values surrounding food and feeding practices. Consequently, individuals in a community often adopt longstanding and accepted eating habits (Higgs 2015). Maternal social environments, as social determinants of health, are implicated in the adoption or resistance of recommended complementary feeding practices (Herman et al. 2023). Peer support is a mutual support system in which individuals with shared experiences encourage and provide guidance to one another. It has become popular in creating strong and supportive communities for breastfeeding and complementary feeding. Drawing on their lived experiences as caregivers, peer support members offer emotional support (including feelings of belonging and acceptance), instrumental support (practical assistance with feeding), informational support (knowledge and guidance), and appraisal support (constructive feedback) (Evans 2023; Keats et al. 2021). While these peer support dimensions are valuable, they cannot substitute for addressing structural barriers, such as poverty and food insecurity, which remain the primary barriers to complementary feeding practices (Deeney and Harris‐Fry 2020).

Peer support builds social capital, encouraging participation in personalised or group nutrition activities, thereby fostering a supportive platform that promotes behavioural changes (Haldane et al. 2019). However, the effectiveness of peer support in promoting complementary feeding practices varies. According to collective action theory (Oberschall 2023), for peer support to be effective, it requires women to come together to adopt recommended child‐feeding practices that they may not be familiar with or may not be able to achieve on their own. The theory addresses the problem of ‘free‐riding’, where members benefit from the group but do not contribute, requiring them to contribute at least their time by attending sessions; thus, a sense of ownership and accountability are key to group motivation and cohesion (DeMarrais and Earle 2017).

Peer support is increasingly recognised as a key community‐led collaborative effort that empowers communities to take ownership of improving infant and young child feeding, as recognised by UNICEF (UNICEF 2024). However, a previous synthesis of evidence on peer support interventions to promote complementary feeding has focused primarily on their effectiveness. For instance, recent reviews in LMICs revealed that children of mothers who engaged in peer support had shifted to recommended feeding practices, resulting in a higher rate of children starting solid and semisolid foods between 6 and 8 months, and improved rates of meeting MMF and MDD. However, the underlying mechanisms of behavioural change remain understudied (Haque et al. 2023; Janmohamed et al. 2020; Watson 2019). Furthermore, these systematic reviews were not theory‐led and did not adequately address why and how peer‐to‐peer learning should be institutionalised and sustained (Glasgow 2013; Herman et al. 2023).

Linking peer support group membership to better infant and young child nutrition is a minimalist approach, as it overlooks the complexities of group dynamics and the context‐specific realities of resource‐limited settings. A realist review of peer support interventions necessitates examining them in the context of what works, for whom, and under what specific circumstances. Synthesising evidence via a theoretical lens enhances the practicality and real‐world relevance of evidence that supports decision‐making, such as the integration of peer support within the broader system (Walker and Peterson 2020). The previous review identified factors influencing the effectiveness of peer support, highlighting both practical challenges and opportunities (Haque et al. 2023; Janmohamed et al. 2020). To address gaps in understanding how peer support programmes were implemented, we conducted a theory‐informed systematic review.

Normalisation process theory (NPT) (McNaughton et al. 2020) provides a framework for examining the integration and establishment of peer support within routine healthcare practices. We focus on one of the four constructs of the NPT: cognitive participation, which provides a framework for understanding the relational work that people undertake to build and sustain a community of practice centred on peer support, which includes the commitment and engagement of individuals to support the implementation process (Huddlestone et al. 2020; May et al. 2018). This involves having the right skills for initiation (joining), maintaining commitment (enrollment), validating efforts (legitimisation), and ongoing peer support (activation) (McNaughton et al. 2020). Therefore, this theory‐led systematic review aimed to understand why people choose to participate in, support, and continue using peer support for complementary feeding practices. We examined how participants came to accept and commit to it and how it became a regular part of the complementary feeding practices support system.

## 2 Methods

**Figure 1:**
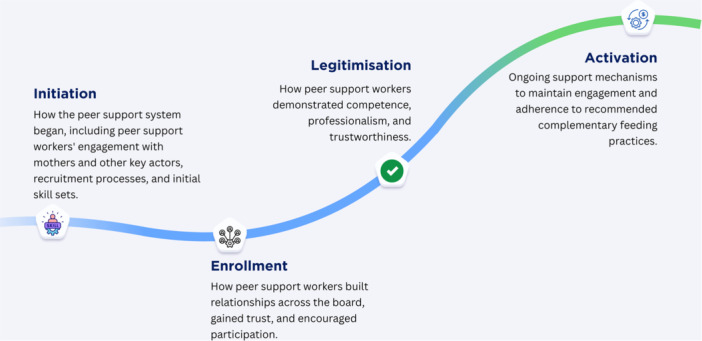
Operational definitions of the four subconstructs of cognitive participation of NPT as applied in peer support evidence synthesis.

The PICO (population, intervention, outcome, comparison) statement of this review was based on the cognitive participation construct of NPT. Building on this framework, we conducted a theory‐informed systematic review using the four subconstructs of cognitive participation, as shown in Figure 1.

***Inclusion and Exclusion Criteria***

The inclusion and exclusion criteria are summarised in Table 1. This review examined studies on peer support for complementary feeding practices, focusing on how peer support is organised, the conditions affecting success, and the target populations.

**Table 1:** Inclusion and exclusion criteria.

Criteria	Inclusion criteria	Exclusion criteria
Types of studies	RCTs and quasi‐experimental, programme evaluation and mixed‐method studies.	All other designs
Population/context (P)	Infants and young children 6‐23 months in Low and middle‐income countries (LMICs)	Other age ranges and contexts. We excluded studies that enroled individuals with special needs, such as those with HIV/AIDS and other illnesses and contexts like nutrition emergencies.
Intervention/exposure (I)	Peer support intervention aimed at improving complementary feeding practices with primary delivery modes of interpersonal communication. Peers are women with relatable experiences in child feeding, facilitating peer support through one‐on‐one or group interactions involving diverse activities.	Other peer support interventions are delivered virtually or through hybrid models, including mobile messages, chatbots, and peer support hotlines.
Comparison	Usual care model	
Outcomes (O)	Four NPT‐defined outcomes (Figure 1) with a focus on cognitive participation in peer support for complementary feeding: willingness to engage, ongoing commitment, and validating and sustaining engagement. Studies were included if they provided evidence reflecting one or more dimensions of cognitive participation as operationalised in our synthesis framework.	
Timeframe	1990–2025	
Language	English	All other languages
Geographic location	LMICs	Other contexts in High‐income countries
Publication status	Peer‐reviewed published articles, associated grey literature sources and published protocols	Articles without full text; conference proceedings

### 2.1 Search Strategy and Information Source

The search period, from January 1990 to February 2025, focused on LMICs, as the WHO Biennial Report (1990–1991) promoted community involvement in health, and prioritised proper food and nutrition as one of the five priority areas (World Health Organization 1992). Subsequent initiatives, such as the Baby Friendly Hospital Initiative (BFHI), incorporated maternal support and counselling (Gomez‐Pomar and Blubaugh 2018). The search strategy employed keywords and MeSH terms related to peer support, complementary feeding practices, and geographic location, and applied filters for publication date, study type, and language. The full line‐by‐line search strategy is outlined in Supporting Information S1: Table 1 (SF1). The following databases were searched: CINAHL Complete, Web of Science, Embase, Scopus, ProQuest Dissertations & Theses, the Cochrane Library, and Google Scholar. The review was supplemented by a manual search of reference lists from included articles, published intervention protocols, grey literature, and relevant websites of international and national organisations.

### 2.2 Selection of Studies

**Figure 2:**
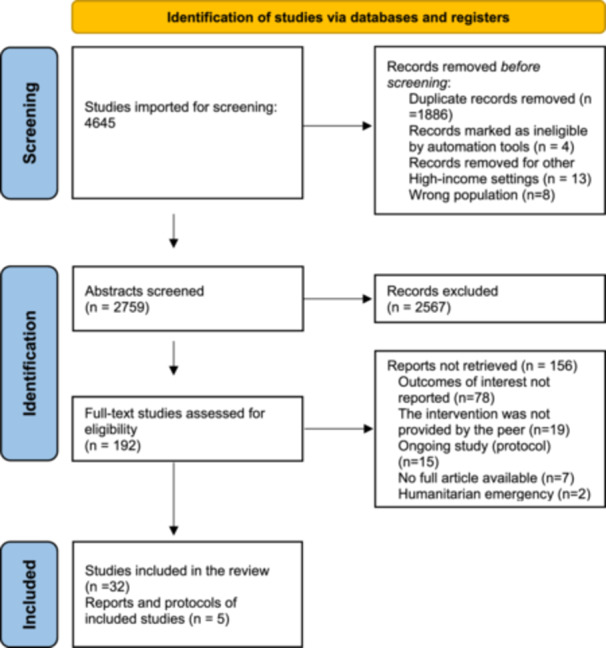
PRISMA flow diagram for systematic reviews.

Initial article screening was conducted in Covidence (Veritas Health Innovation 2023) by two co‐authors, AI and HS, based on title and abstract information. The results were coded as ‘yes’, ‘no’, or ‘maybe’. Articles coded as ‘yes’ were retrieved for full‐text review. Co‐authors carefully reviewed articles rated as ‘maybe’, which underwent full‐text review when agreed upon. AI and HS conducted a full‐text review, and their differences were resolved via discussion (QSR International 2023). Following the updated PRISMA guidelines for reporting systematic reviews, the results of this screening process are shown in Figure 2 (Page et al. 2021).

The initial search yielded 4654 records. After identifying and removing exact duplicates based on authors, title, and year of publication in EndNote (Clarivate 2023), 2759 records remained for abstract screening. These articles were then uploaded to Covidence for further removal of partial duplicates from reports, conference proceedings, or preprints of similar studies. As a result, a total of 192 studies were assessed for full‐text review, and ultimately, 32 studies were included in the final review.

### 2.3 Quality Assessment

The quality and overall strength of the evidence were assessed using the Academy of Nutrition and Dietetics Quality Criteria checklist (Academy of Nutrition and Dietetics 2016). We assessed the risk of bias of each article using this criteria checklist, and each study was classified as having a high, low, or unclear risk of bias. We incorporated these assessment observations into our results and discussion sections. The full report is provided in Supporting Information S1: Table 2 (SF2).

### 2.4 Data Extraction and Evidence Synthesis Approach

**Figure 3:**
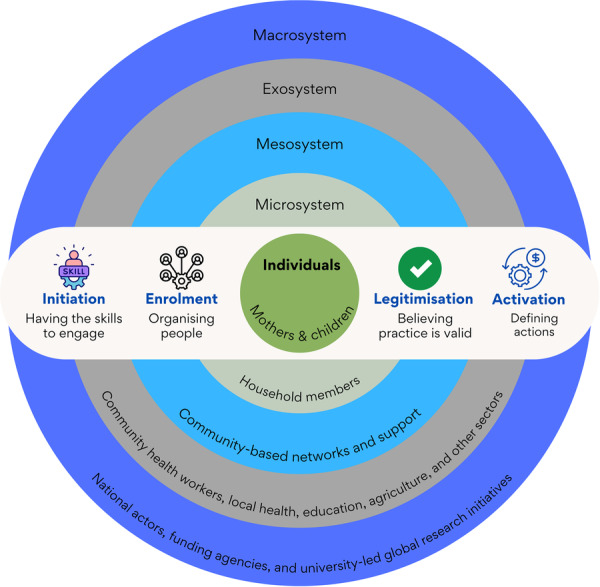
Illustration of the cross‐sectional nature of cognitive participation across multilayered actors.

This pragmatic theory‐led review employed an integrative mixed‐methods synthesis. We identified stakeholders as all actors mentioned in the studies as involved in or affected by peer‐led complementary feeding interventions. These stakeholders were then systematically mapped using the ecological model shown in Figure 3, which organises them by their level of influence. This approach established the complex network of actors involved in peer support programmes. The synthesis incorporates quantitative findings and textual descriptions throughout the methods, results, and discussion. AI and HS utilised operational definitions (Figure 1) of initiation, enrollment, legitimation, and activation to code full‐text articles. This process grouped them into common and unique themes and examined how these themes change across subconstructs. These are presented separately as barriers and facilitators. We extracted numerical data and included it as a range to account for real‐world variability across contexts, populations, and implementation strategies. The methodological quality and risk of bias were systematically evaluated, with insights from these assessments integrated throughout the results (SF2).

## 3 Results

This review included 32 articles, their associated grey literature, and published protocols associated with the peer‐reviewed articles. The studies were conducted in 12 countries; Bangladesh (*n* = 9) had the most studies, followed by India (*n* = 8), Nepal (*n* = 4), and Ethiopia (*n* = 2). Other countries, including Nigeria, Malawi, Kenya, Rwanda, Burkina Faso, Ecuador, China, Vietnam, and Tajikistan, each had one study. All studies were conducted in rural areas, except one in Nairobi, Kenya. The studies covered five WHO regions: Southeast Asia (*n* = 21), Africa (*n* = 7), the Western Pacific (*n* = 2), the Americas (*n* = 1), and Europe (*n* = 1).

The studies included cluster‐randomised controlled trials (*n* = 13) (Acharya et al. 2019; Arifeen et al. 2009; Flax et al. 2022; Flax et al. 2021; Kim et al. 2018; Kim et al. 2019; Menon et al. 2016; Olney et al. 2015; Rawat et al. 2017; Saville et al. 2018; Shi et al. 2010; Warren et al. 2020; Younes et al. 2015), quasi‐experimental studies (*n* = 14) (Komal et al. 2014; Mondal et al. 2023; Mukuria et al. 2016; Newton‐Lewis and Bahety 2021; Nguyen et al. 2019; Rahman et al. 2023; Roche et al. 2017; Saggurti et al. 2018; Singh et al. 2017; Singh et al. 2017; Suresh et al. 2019; Vir et al. 2014; Yorick et al. 2021); mixed‐methods process evaluations (*n* = 2) (Kim et al. 2015; Sanghvi et al. 2016; Scott et al. 2018); programme implementation evaluation (*n* = 1) (Sanghvi et al. 2016); observational study using Lot Quality Assurance Sampling (LQAS) (*n* = 1) (Das et al. 2016), and qualitative studies (*n* = 1) (Jahir et al. 2021).

### 3.1 Stakeholders Involved

All studies involved community‐based interventions. Our stakeholder analysis revealed multiple layers of actors with diverse roles, as outlined in Table 2. This table summarises how cognitive participation intersects across the socioecological framework (Figure 2), with a particular focus on mothers and children (Table 2).

**Table 2:** Socioecological classification of all actors involved in the studies.

Study	Country	Targets groups (direct beneficiary)	Microsystem (family members)	Mesosystem (community‐based actors)	Exosystem (local health and research actors)	Macrosystem (international bodies)
Acharya et al. (2019)	Nepal	Mothers, infants, and pregnant women	Family members (Conveyed mobile text messages to the participants in the intervention arm)	Female Community Health Volunteers (FCHVs) (Provided information about maternal and child health care services to pregnant women)	Researchers, District Public Health Office, Dhanusha, Nepal; Nepal Health Research Council; and Janaki Medical College Council	Institute of Medical Sciences, Banaras Hindu University, India
Arifeen et al. (2009)	Bangladesh	Mothers and children under 5 years	Other caretakers (Provided data on exposure to community‐based interventions)	Village Health Workers (VHWs) (Provided community case management of nonsevere pneumonia and diarrhoea); Village Doctors (Trained to avoid harmful treatment practices and refer severely ill children); Community Nutrition Promoters (CNPs) (Conducted household counselling and mothers' group meetings); Imams (Trained to convey messages about IMCI during Friday prayer sermons)	Physicians (Observed case management by VHWs)	World Health Organisation (WHO), UNICEF, Bill & Melinda Gates Foundation, WHO's Department of Child and Adolescent Health and Development, and USAID (Funding)
Das et al. (2016)	India	Mothers and children (9–11 months) (exposure to FLW services on infant feeding practices)		Frontline Workers (FLWs) (Provided nutritional counselling services to mothers)Anganwadi Workers (AWWs) (Provided essential health care services and nutritional counselling); Accredited Social Health Activists (ASHAs) (Provided community health services and nutritional counselling)	BRAC (Implemented the IPC and CM activities); Auxiliary Nurse Midwives (ANMs) (Provided maternal and child health services and nutritional counselling)	Bill and Melinda Gates Foundation (Funding)
Flax et al. (2022)	Nigeria	Mothers and children 0–23 months	Families (Received IYCF messages and support)	Community Volunteers (Conducted home visits and community meetings to discuss IYCF); Religious and Community Leaders (Participated in community mobilisation efforts)	Health Workers (Provided IYCF counselling and support at health facilities); Kaduna State Ministry of Health and Lagos State Government; Alive & Thrive (Implemented the IYCF intervention); RTI International (Conducted the evaluation); Institutional Review Boards at RTI and FHI 360	Bill & Melinda Gates Foundation, Irish Aid, Tanoto Foundation, UNICEF, and World Bank (Funding)
Flax et al. (2021)	Rwanda	Mothers and children 12–29 months		Community Health Workers (CHWs) (Conducted household and community SBCC sessions promoting ASF consumption)	National Child Development Agency	Three Stones International and USAID
Gope et al. (2019); Prost (2019)	India	Mothers and children under 3.		Trained local female workers **(**Facilitated PLA meetings and provided counselling to mothers at home)	Ekjut (Independent ethics approval committee); Public Health Resource Network (PHRN) (Provided ethical approval for the study)	Tata Trusts (Funding)
Jahir et al. (2021)	Bangladesh	Mothers and children under 2 years		Community Health Workers (CHWs) (Implemented the integrated intervention; conducted group meetings and home visits); Supervisors (Supervised CHWs; provided feedback and on‐the‐job training)	ICDDR; The University of California Davis	Bill and Melinda Gates Foundation (Funding)
Kim et al. (2015)	Ethiopia	Mothers and children under 2 years		Frontline Workers (FLWs) (Delivered IYCF interventions to mothers and caregivers); Community Volunteers (Supported HEWs in IYCF activities)	Health Extension Workers (HEWs) (Provided IYCF counselling and education at health posts and in the community); Alive & Thrive (A&T) (Provided training and materials for IYCF interventions)	Bill & Melinda Gates Foundation, and CGIAR Research Programme on Agriculture for Nutrition and Health (A4NH). (Funding)
Kim et al. (2018)	Bangladesh	Mothers and children 0–23.9 months		Community Volunteers (Provided IYCF counselling and support services); Frontline Workers (Provided IYCF counselling and support services); Pushti Kormi (Nutrition Promoters) (Conducted multiple age‐targeted IYCF‐focused visits to households)	Alive & Thrive (A&T) (Provided intensive interventions (IPC, CM, and MM) to improve IYCF practices)	Bill & Melinda Gates Foundation and CGIAR Research Programme on Agriculture for Nutrition and Health (A4NH) (Funding)
Kim et al. (2019)	Ethiopia	Mother and children 6–23.9 months		Health Development Team Leaders (HDTLs) (Provided IYCF messaging during home visits); Ethiopian Orthodox Church Priests/Leaders(Delivered CM activities, such as sermons about child feeding during religious fasting periods)	Health Extension Workers (HEWs) (Provided IYCF counselling during health post and home visits, and conducted food demonstrations); Agricultural Extension Workers (Promoted nutrition‐sensitive agricultural activities); Alive & Thrive (A&T) (Provided intensive interventions (IPC, AG, CM, and MM) to improve IYCF practices)	Bill & Melinda Gates Foundation (Funding); Governments of Canada and Ireland (Funding); CGIAR Research Programme on Agriculture for Nutrition and Health (A4NH) (Funding)
Komal et al. (2014)	India	Mothers, infants and young children	Mother Support Groups (MSGs) (Provided counselling and support to mothers on IYCF practices)	Anganwadi Workers (AWWs) (Served as members of MSGs); ASHAs (Community Health Workers) (Served as members of MSGs); Dais (Traditional Birth Attendants) (Served as members of MSGs); Local Leaders (Coordinated in identifying potential candidates for MSGs); Primary School Teachers (Coordinated in identifying potential candidates for MSGs)	Dept of Paediatrics, BRD Medical College, Gorakhpur; Integrated Child Development Schemes (ICDS) (Coordinated in identifying potential candidates for MSGs.)	UNICEF (Funding)
Menon et al. (2016)	Bangladesh	Mothers, children 6–23.9 months	Families (engaged in supporting IYCF behaviours)	Community Leaders (to IYCF); Shasthya Sebika (Community Volunteer) (Conducted routine home visits and provided information on IYCF practices); Pushti Kormi (Nutrition Promoters) (Conducted multiple age‐targeted IYCF‐focused counselling visits to households); Shasthya Kormi (Health Workers) (Conducted routine home visits and provided information on IYCF practices);	BRAC (Implemented the IPC and CM activities); Alive & Thrive (A&T) (Provided intensive interventions (IPC, CM, and MM) to improve IYCF practices)	Bill & Melinda Gates Foundation and CGIAR Research Programme on Agriculture for Nutrition and Health (A4NH**)** (Funding)
Mondal et al. (2023)	India	Mothers and children 6–23 months		JEEVIKA Community Mobilisers (Conducted BCC module roll‐out in SHG meetings), JEEVIKA (Facilitated the roll‐out of the BCC module)	AIIMS Patna Ethics Committee and SIGMA Institutional Review Board, Government of Bihar (Approved the Parivartan initiative); Project Concern International (PCI) (Developed the BCC module and collected data)	Bill and Melinda Gates Foundation (BMGF) (Funding)
Mukuria et al. (2016)	Kenya	Mothers and infants 6–18 months	Fathers (Participated in dialogue groups and provided social support to mothers); Grandmothers (Participated in dialogue groups and provided social support to mothers).	Community Health Workers (CHWs) (Supported the intervention and monitored group activities); Dialogue Group Mentors (Facilitated discussions and activities in the dialogue groups)	Ministry of Health (MOH) (Provided guidance on study site selection); Community Health Extension Workers (CHEWs) (Oversaw the work of CHWs and dialogue groups); AIDS, Population and Health Integrated Assistance Plus (APHIAplus) Western Kenya Project (Provided guidance on study site selection); PATH Research Ethics Committee; The Kenyatta National Hospital/University of Nairobi Ethics and Research Committee	USAID (Funding)
Newton‐Lewis and Bahety (2021)	India	Mothers and children 3–23 months		Accredited Social Health Activists (ASHAs) (provided home visits delivering health services and education)	Sambodhi Research and Communications; Public Healthcare Society (India).	Norway India Partnership Initiative (NIPI), Norad and Bill and Melinda Gates Foundation (Funding)
Nguyen et al. (2019)	Bangladesh	Mothers and Children (0–23.9 months)	Families (Engaged in information sharing and support for IYCF practices)	Community Volunteers (Provided standard information on IYCF practices during home visits); Pushti Kormi (Nutrition Promoters) (Conducted age‐targeted IYCF‐focused visits in intensive areas); BRAC Frontline Workers (Provided standard information on IYCF practices during home visits)	Alive & Thrive (A&T) (Implemented intensive interventions (IPC, CM, and MM) to improve IYCF practices)	Alive & Thrive (A&T) (Implemented intensive interventions (IPC, CM, and MM) to improve IYCF practices) Bill & Melinda Gates Foundation and CGIAR Research Programme on Agriculture for Nutrition and Health (A4NH) (Funding)
Olney et al. (2015)	Burkina Faso	Mothers and children (3–12.9 months at baseline)	Older Women Leaders (OWLs) (Delivered BCC messages in one treatment group)	Health Committee (HC) Members (Delivered BCC messages in another treatment group)	Ministry of Health of Burkina Faso; Helen Keller International (HKI) (Implemented the E‐HFP programme); International Food Policy Research Institute and Michigan State University	US Agency for International Development (USAID), Gender, Agriculture and Assets Project (GAAP), CGIAR Research Programme on Agriculture for Nutrition and Health (A4NH) and National Institute of Food and Agriculture (USDA) (Funding)
Rahman et al. (2023)	Bangladesh	Children (6– 23 months) and caregivers		Shasthya Shebikas (Female Volunteer CHWs) (Provided primary healthcare services and distributed MNP sachets)	BRAC (Implemented the Maternal, Infant and Young Child Nutrition programme (MIYCN); ICDDR (Evaluated the MIYCN programme)	Children's Investment Fund Foundation (CIFF) (Funding); Global Alliance for Improved Nutrition (GAIN), Renata Pharmaceutical Company and Social Marketing Company (SMC) (Partnered in the MIYCN programme
Rawat et al. (2017)	Vietnam	Mothers, Children (6–23.9 months) and Children (24–59.9 months)		Village Health Workers (Delivered invitation cards, encouraged mothers to attend MTBT counselling services, and provided essential IYCF messages in intensive areas)	Government Health Facilities (Provided IYCF counselling services under the MTBT brand); Save the Children (Implemented the social franchise model); Alive & Thrive (A&T) (Supported the social franchise model and MM campaign)	Bill & Melinda Gates Foundation (Funding) CGIAR Research Programme on Agriculture for Nutrition and Health (A4NH) (Funding)
Roche et al. (2017)	Ecuador	Mothers and children under 2 years		Community Leaders (Selected comparison communities, nominated Madre guias (volunteer peer educators); Madre Guias (Volunteer Peer Educators**)** (Led PD/Hearth cooking and nutrition education sessions; conducted home visits)	World Vision Ecuador (Implemented the PD/Hearth intervention); Ministerio de Salud Pública Dirección de Nutrición; McGill University Faculty of Medicine Institutional Review Board	International Development Research Centre of Canada, Canadian Institutes for Health Research and World Vision Canada (Funding)
Saggurti et al. (2018)	India	Mothers (Members of SHGs) and children 0–12 months		Women's Self‐Help Group (SHG) Members (Participated in group discussions and activities; provided peer support and information sharing)	Community Health Facilitators (Sahelis) (Delivered the health intervention and facilitated participatory training on MNCH issues); Population Council (Designed and conducted the study)	Bill and Melinda Gates Foundation and Project Concern International (Funding)
Sanghvi et al. (2016)	Bangladesh	Mothers and children 0–23 months	Families (Engaged in supporting IYCF behaviours)	Community leaders (Sensitised to IYCF); Shasthya Sebika (Community Volunteer) (Conducted routine home visits and provided information on IYCF practices); Pushti Kormi (Nutrition Promoters) (Conducted multiple age‐targeted IYCF‐focused counselling visits to households); Shasthya Kormi (Health Workers) (Conducted routine home visits and provided information on IYCF practices)	BRAC (Implemented the IPC and CM activities); Alive & Thrive (A&T) (Provided intensive interventions (IPC, CM, and MM) to improve IYCF practices)	Alive & Thrive (A&T) (Provided intensive interventions (IPC, CM, and MM) to improve IYCF practices) Bill & Melinda Gates Foundation and CGIAR Research Programme on Agriculture for Nutrition and Health (A4NH) (Funding)
Saville et al. (2018)	Nepal	Mothers and children 0–16 months	Husbands (Their vasectomy status was considered for women's eligibility)	Female Community Health Volunteers (FCHVs) (Facilitated PLA women's groups delivered usual government outreach services in the control arm); Supervisors (Supported FCHVs and mobilisers, monitored intervention activities); Nutrition Mobilisers (Assisted with group facilitation, transfer distribution, and record‐keeping)	Mother and Infant Research Activities (MIRA)(Implemented the study); Nepal Health Research Council (Data Monitoring Committee (DMC); Trial Steering Committee); University College London (UCL)	UKaid from the Department for International Development, South Asia Research Hub, Economic and Social Research Council (ESRC), Department for International Development (DFID), Wellcome Trust, Medical Research Council (MRC) (Funding)
Scott et al. (2018)	Malawi	Mothers and children 0–53 months	Husbands (Provided data on extended family members, participated in key‐informant interviews), Grandmothers (Maternal and Paternal) (Their presence and influence on child health and intervention effectiveness were examined)	Peer Counsellors(Trained local women volunteers; provided home visits to mothers; delivered information on infant care and nutrition); Village Chiefs (Participated in key‐informant interviews); Community Health Workers (CHWs) (Provided mentorship and supervision support to peer counsellors; participated in key‐informant interviews)	National Health Sciences Research Committee, Malawi; Oxford Policy Management (Conducted the study)	ESRC/DFID (Funding)
Shi et al. (2010)	China	Caregivers and infants 2–12 months	Husbands (provided a supportive environment for changing feeding practices), Parents‐in‐law (provided a supportive environment for changing feeding practices)	Village Committee Leaders (Participated in group training sessions on complementary feeding); Village Doctors (Participated in group training sessions on complementary feeding)	Local healthcare providers (Delivered the educational intervention, conducted surveys, and measured anthropometrics); Johns Hopkins Bloomberg School of Public Health and Peking University Health Science Centre	Proctor & Gamble Fellowship (Funding)
V. Singh et al. (2017)	India	Mothers and children 0–18 months		Anganwadi Workers (AWWs) (Provided nutrition and health care education, conducted home visits); Auxiliary Nurse Midwives (ANMs) (Provided antenatal care, delivery care, and immunisation services)	King George Medical University (KGMU)(Collaborated on the study); Cooperative for Assistance and Relief Everywhere (CARE) (Implemented the enhanced nutrition and health programme)	Johns Hopkins Bloomberg School of Public Health (JHBSPH) (Designed and implemented the study); USAID (Funding); India Mission, Gates Grant GH 614, and George G. Graham Professorship Endowment (Support study)
A. Singh et al. (2017)	Nepal	Mothers and children 6–23.9 months		Peer Facilitators (PFs) (Provided support and education to mothers on MIYCN practices)	Suaahara (Implemented the integrated nutrition programme); Helen Keller International (Led the implementation of Suaahara)	USAID (Funding)
Suresh et al. (2019)	Nepal	Mothers and children under 5 years	Community Members (Participated in SII community mobilisation events)	Suaahara II (SII) (Implemented the multisectoral nutrition programme); Frontline Workers (FLWs) (Conducted home visits and provided counselling and support to mothers)	New ERA (Conducted the annual monitoring survey for SII); Nepal Health Research Council (NHRC); Helen Keller International (HKI) (Led the implementation of SII)	USAID (Funded the SII programme)
Vir et al. (2014)	India	Mothers and children under 2 years	Family (received counselling and support from mitanins)	Mitanins (Community Health Volunteers) (Conducted family‐level counselling; mobilised the community to improve coverage of maternal and child health services; promoted complementary feeding and PDS entitlement)	Public Health Nutrition and Development Centre (Led the study); The Centre for Child Health and Nutrition (Provided ethical clearance for the study); Health Department (Initiated the Mitanin Programme), Integrated Child Development Services (ICDS) (Coordinated with mitanins to improve coverage of nutrition interventions); Public Distribution System (PDS) (Provided access to subsidised food items, promoted by mitanins); National Rural Health Mission (NRHM) (Provided incentives to mitanins)	The ICICI Foundation for Inclusive Growth supported the study.
Warren et al. (2020)	Bangladesh	Mothers and children under 3 months	Families (Received BCC messages on IYCF)	Community Volunteers (Shasthya Sebika) (Conducted routine home visits; provided information on IYCF practices in nonintensive areas) BRAC Frontline Workers (Shasthya Kormi) (Conducted routine home visits; provided information on IYCF practices in nonintensive areas) Pushti Kormi (Nutrition Promoters) (Conducted age‐targeted IYCF‐focused visits in intensive areas)	Alive & Thrive (Implemented the BCC intervention); International Food Policy Research Institute and the Bangladesh Medical Research Council.	Bill & Melinda Gates Foundation and CGIAR Research Programme on Agriculture for Nutrition and Health (A4NH) (Funding)
Yorick et al. (2021)	Tajikistan	Mothers and children under 5 years	Families (received education and support from CHWs and CAWs)	Community Health Workers (CHWs) (Provided MNCH services; promoted nutrition and WASH; conducted household visits; referred children with malnutrition and pregnant women to health facilities); Community Agricultural Workers (CAWs) (Reinforced agricultural practices; promoted maternal and child health and nutrition; conducted household visits and community events).	Rural Health Facilities (Provided health services; received referrals from CHWs); Government Healthy Lifestyle Centres (HLSCs); (Coordinated the work of CHWs; participated in training and community events); IntraHealth International (Implemented the THNA project).	USAID (Funding)
Younes et al. (2015)	Bangladesh	Mothers and children under 5 years	Families (Participated in the women's groups and community meetings)	Women's Group Members (Participated in group discussions and activities, contributed to community mobilisation efforts), Community Members (Participated in community meetings and engaged in discussions on child health issues and strategies); Facilitators (Led the women's groups, providing training and support to group members); Supervisors (Supported facilitators, liaising with community leaders and healthcare providers).	Diabetic Association of Bangladesh (BADAS) (Implemented the study in collaboration with UCL); Local healthcare providers (government and nongovernmental); (Involved in training and community mobilisation efforts); Community clinic committees, union council health committees, upazila health advisory committees, and upazila health and family planning coordination meetings (Participated in health systems strengthening initiatives); Great Ormond Street Hospital and Institute of Child Health (GOSH‐ICH); UCL Institute for Global Health (Collaborated on the study design and implementation).	The Big Lottery Fund (UK), through an International Strategic Grant (Funding)

Further findings from our stakeholder mapping (Supporting Information S1: Table 1) revealed differences in who engaged in peer support. Few studies have reported family members as active participants in interventions that would enable cognitive participation, and these have been limited to the health sector delivery platforms. Furthermore, the interventions were donor‐funded and implemented through partnerships between donors and research organisations in the Global North and implementing organisations in the Global South.

### 3.2 Facilitators of Peer Support

**Figure 4:**
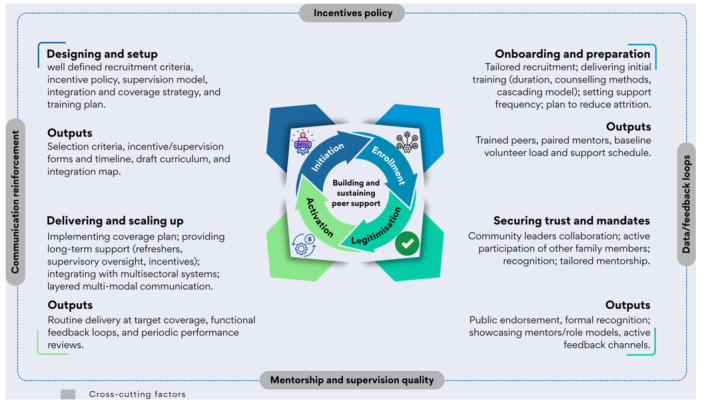
Factors influencing peer support for complementary feeding practices through the NPT lense.

The initiation of peer support for complementary feeding involved recruiting volunteers who met specific selection criteria. Common recruitment criteria included local credibility, experience in maternal and child health, maternal educational status, age, and willingness to participate (Gope et al. 2019; Jahir et al. 2021; Komal et al. 2014; Mukuria et al. 2016; Saggurti et al. 2018; Shi et al. 2010; Singh et al. 2017; Vir et al. 2014; Yorick et al. 2021). This process and efforts to build participants' interest and commitment established the legitimacy of peer support among communities. As a result, the early implementation phase was time‐ and resource‐intensive. The alignment of peer support activities with health systems has, to a certain extent, enhanced the scaling and continuity of peer support systems (Figure 4). The implementation, from its inception to activation, does not follow a sequential progression through initiation, enrollment, legitimisation, and activation. Instead, these stages overlap and interact with one another. The priorities were to gain community trust and involvement by identifying and recruiting suitable volunteers, thereby promoting sustainability. We organised the facilitators into common themes as follows.

#### 3.2.1 Structured Training and Capacity Building

Peer support training covered elements such as content delivery, skills demonstration, and ongoing supervision and support. Training across studies lasted between 2 and 8 days. Effective training was built on suitable candidates who could deliver locally tailored skills and were available for structured upskilling sessions. Initial training sessions focused on equipping peer support workers with the core skills necessary to promote improved feeding practices. Training prioritised tailored communication skills for building rapport with mothers and providing culturally appropriate counselling techniques. Several studies have emphasised the importance of providing intensive support during early implementation stages (Flax et al. 2022; Jahir et al. 2021; Komal et al. 2014; Mukuria et al. 2016; Singh et al. 2017; Yorick et al. 2021).

Several studies described a cascaded training model, as in the Alive and Thrive initiative across Bangladesh, Ethiopia, and Vietnam, where health workers first received training and then trained peer counsellors (Kim et al. 2015; Menon et al. 2016; Rawat et al. 2017; Sanghvi et al. 2024). Even in such structured training, volunteers required continued skill reinforcement through refresher training, supervision, and mentorship. In Tajikistan, these efforts, particularly clarifying on role definitions and strong mentorship, reduced volunteer dropout rates from 17% to just 1% (Yorick et al. 2021). Conversely, inadequate post‐training support in other settings resulted in lower implementation fidelity and diminished retention of best practices (Kim et al. 2015; Komal et al. 2014; Singh et al. 2017). For more details about training and skill retention challenges, see Section 3.3.2.

#### 3.2.2 Community Engagement and Ownership

Multiple studies have shown that involving community gatekeepers, such as fathers, grandmothers, religious authorities, and other community figures, increased acceptance of and commitment to peer support interventions (Kim et al. 2015; Menon et al. 2016; Mukuria et al. 2016; Nguyen et al. 2019). For instance, a study from Kenya showed that community leaders' involvement enhanced the initial uptake and commitment of mothers to the adoption of feeding recommendations. Additionally, engaging father and grandmother strengthened the family support system (Mukuria et al. 2016). Building on this, mothers who joined established peer support networks showed consistent attendance of intervention sessions and perceived greater programme credibility through community endorsement (Kim et al. 2015; Mukuria et al. 2016; Nguyen et al. 2019). Collectively, these social and family validations, when combined with practical peer‐led advice, contributed to improvements in MDD, ranging from approximately 7 to 28 percentage points (Kim et al. 2015; Kim et al. 2019; Mondal et al. 2023; Mukuria et al. 2016; Nguyen et al. 2019; Rawat et al. 2017; Singh et al. 2017).

#### 3.2.3 Incentives and Supervision

Both financial and non‐financial incentives have improved the recruitment and retention of peer support workers (Kim et al. 2018; Nguyen et al. 2019; Yorick et al. 2021). Financial incentives vary across studies and contexts. In Bangladesh, monthly payments ranged from $6 to $8 per month (Kim et al. 2018; Nguyen et al. 2019) and went up to $35 (Jahir et al. 2021). Additional material incentives, such as blankets, umbrellas, and small prizes (Yorick et al. 2021), were also provided. Non‐monitory and non‐material recognitions were also important. Yorick et al. (2021) found that recognition programmes, including certificates, public acknowledgement and continued training, reduced peer support workers attrition from 17% to 1% (Yorick et al. 2021), thus maintaining the continuity of support (Kim et al. 2018; Yorick et al. 2021).

Performance‐based incentives contributed to the retention of peer support workers, as seen in improved behaviour: participating households were up to 1.3–4.1 times more likely to meet MDD in Bangladesh, India, Ethiopia, and Nepal (Kim et al. 2015; Kim et al. 2018; Kim et al. 2019; Mondal et al. 2023; Suresh et al. 2019). However, challenges remain: monetary incentives often lack long‐term viability due to short‐term donor funding and limited financial flexibility for maintaining incentives, leading to reduced performance‐based incentives and peer support worker attrition (Kim et al. 2018; Yorick et al. 2021), as detailed in Section 3.3.1.

Beyond financial and material rewards, volunteers value community recognition and respect from the families they serve. The Mitanins Programme in India showed how linking specific tasks to recognition and support maintained volunteer commitment (Vir et al. 2014). Some interventions recognised successful mothers as role models. For instance, India's PARU recognition ceremony was organised to honour mothers who met the MDD, enabling them to become role models, as demonstrated in the Purak Ahaar Ratna Utsav (PARU) recognition ceremony in India (Mondal et al. 2023). Regular supportive supervision improved retention by helping workers feel valued in their role. In India, Kenya and Ethiopia, regular supervision improved retention and adherence to established protocols (Kim et al. 2015; Komal et al. 2014; Mukuria et al. 2016).

#### 3.2.4 Multimodal Strategies and Coverage

Community‐based peer support programmes used various delivery approaches, with coverage varying across contexts. Programmes that integrated peer facilitators into the existing health system reported coverage rates from 28% to 60% (Singh et al. 2017). This variation reflected how well each peer system leveraged local strengths and contexts to optimise resources and avoid duplication of effort. Integrated multimodal approaches, combining interpersonal communication, community mobilisation and mass media, improved complementary feeding practices (Kim et al. 2019; Menon et al. 2016). Furthermore, programmes that utilised multiple contact points, such as combining home visits with group sessions, have shown improvement in complementary feeding practices compared to single approaches (Nguyen et al. 2019; Roche et al. 2017; Suresh et al. 2019; Younes et al. 2015). For example, the multimodal approach yielded improvements of 16.3 percentage points in MDD and 14.7 percentage points in MMF in intensive intervention areas of Bangladesh, and significant improvements were documented in Vietnam (Menon et al. 2016; Rawat et al. 2017).

Studies consistently linked regular and frequent contact, whether through home visits or group meetings, to improved programme participation (Singh et al. 2017; Younes et al. 2015). For instance, an Indian study found that more frequent and higher quality contacts improved feeding practices (Singh et al. 2017). These intensive contact strategies increased the timely introduction of complementary feeding practices by up to 12–17 percentage points (Kim et al. 2018; Nguyen et al. 2019; Roche et al. 2017; Younes et al. 2015). These findings suggested the need for ongoing institutional support to sustain the integration of peer support programmes within healthcare systems (Menon et al. 2016; Saville et al. 2018; Shi et al. 2010; Singh et al. 2017; Suresh et al. 2019).

### 3.3 Barriers to Peer Support for Complementary Feeding Practices

Peer support for complementary feeding practices faced several challenges. Family members were sometimes sceptical, and funding was often unreliable. Additional practical issues that affected day‐to‐day functioning and peer session attendance included unclear roles, limited resource allocation, and geographic distance. Misinformation and maternal time constraints further limited attendance at peer sessions. Inadequate compensation and insufficient training undermined trust and credibility in peer support programmes. The lack of sustained momentum of initial activities and weak post‐intervention engagement hindered the effective activation of peer support.

#### 3.3.1 Funding and Resource Constraints

Financial constraints resulting from systemic issues, dependence on external donors, and insufficient governmental support led to operational instability. As illustrated in Table 1, stakeholder mapping revealed that most interventions were driven by multinational organisations, primarily the Bill & Melinda Gates Foundation, USAID, DFID and North‐South research partnerships. Host countries, however, exerted minimal influence over funding priorities and exit strategies. This reliance on external donors and funding transitions contributed to implementation challenges and sustainability issues of the peer support initiatives (Kim et al. 2018; Newton‐Lewis and Bahety 2021; Sanghvi et al. 2016). Several studies have documented the need for ongoing institutional support to sustain the effectiveness of peer support (Menon et al. 2016; Shi et al. 2010).

When external funding ended or was reduced, the peer support programmes experienced a decrease in the intervention exposure. This was evident in Bangladesh, where programme modifications following donor transitions led to reduced intensity and coverage (Kim et al. 2018; Sanghvi et al. 2016). Donors' withdrawal also led to a decline in the retention of community health workers (Acharya et al. 2019; Jahir et al. 2021; Newton‐Lewis and Bahety 2021) and operational challenges that affected complementary feeding support (Kim et al. 2018; Mukuria et al. 2016; Newton‐Lewis and Bahety 2021; Yorick et al. 2021). Specifically, financial difficulties created operational barriers, including shortages of facilitation materials needed for sessions, such as pictorial aids and manuals, a lack of physical venues for meetings, and limited logistical support for organising activities. The inadequate availability of suitable meeting space further reduced participation (Gope et al. 2019; Jahir et al. 2021; Menon et al. 2016; Mukuria et al. 2016; Saggurti et al. 2018; Scott et al. 2018; Singh et al. 2017).

Inadequate compensation, characterised by inconsistent, low, or delayed stipends, undermined the credibility of peer support workers. In India, delayed payments and low stipends ranging from Rs 50 per month (Komal et al. 2014) to variable amounts led to frustration, low morale and dissatisfaction among workers (Newton‐Lewis and Bahety 2021). Across different contexts, inadequate compensation structures contributed to perceived undervaluation and diminished credibility (Jahir et al. 2021; Newton‐Lewis and Bahety 2021). Consequently, less than half of mothers received all scheduled home visits, with coverage as low as 39% in India's HBNC+ programme (Kim et al. 2015; Newton‐Lewis and Bahety 2021; Yorick et al. 2021).

#### 3.3.1 Training and Capacity Building

Training quality and coverage were mixed after a change in national policy. In Ethiopia, from 2011, health extension workers (HEWs) were only to receive training through government‐managed structures, while the concurrent restructuring of community health promoters into the Health Development Army (HAD) was underway. This policy change shortened the intervention to about 1 year, which led to variable training outcomes, including weaker knowledge gains among volunteers in some regions (Kim et al. 2015). There were gaps in the distribution of job aids to volunteers, with only 39%–54% receiving essential tools. As a result, the quality of programme message delivery by volunteers was low, resulting in low maternal message recall and poor aided recall of key messages (Kim et al. 2015). Jahir et al. reported that age‐specific training materials were difficult to deliver (Jahir et al. 2021; Kim et al. 2015; Newton‐Lewis and Bahety 2021). There were missing, postponed or reduced refresher courses, as in Bangladesh, which gradually tapered from monthly to quarterly refreshers before stopping completely in 2016 (Kim et al. 2018). Despite adequate coverage, effective skill‐building remained limited, given that knowledge‐based rather than competency‐based approaches predominated across contexts (Newton‐Lewis and Bahety 2021). As a result, confidence level varied, with community volunteers reporting the lowest self‐efficacy in providing IYCF education and the highest need for further training (92.3% requested additional support) (Kim et al. 2015). There were also session‐completion problems among attendees due to difficulties with session management and multiple intervention components (Jahir et al. 2021).

Large volunteer pools caused supervisory overload. For instance, in Ethiopia, each pair of HEWs supervised 50 volunteers, overwhelming oversight systems. Consequently, only 21.8%–41.8% of volunteers received supervision (Kim et al. 2015), which limited programme reach (Kim et al. 2015; Singh et al. 2017; Yorick et al. 2021). Gaps in training and capacity building had led to decreased exposure to intervention and knowledge of key messages among volunteers, threatening implementation fidelity (Kim et al. 2015; Singh et al. 2017). These supervisory challenges affected volunteer retention and confidence, with programmes demonstrating that improved training and supportive supervision, as in Tajikistan (Yorick et al. 2021), and enhanced community health workers' confidence in carrying out their responsibilities in Bangladesh(Jahir et al. 2021). For details on how structured training supported implementation, see Section 3.2.1.

#### 3.3.2 Community and Sociocultural Barriers

Household gatekeepers, particularly fathers and grandmothers, often questioned the value of peer‐led programmes. Scott et al. (2018) found that Malawian paternal grandmothers actively opposed interventions, telling the mothers that counsellors were ‘cheating you’, which hindered programme acceptance (Scott et al. 2018). Research by Jahir et al. (2021) found that mothers placed greater trust in health professionals than peer supporters (Jahir et al. 2021). This preference created resistance to programme adoption and the promotion of improved complementary feeding practices (Mukuria et al. 2016; Nguyen et al. 2019; Scott et al. 2018).

Targeting educational efforts toward grandmothers reduced resistance to recommended feeding practices. Mukuria et al. (2016) and Scott et al. (2018) reported that these approaches helped align traditional family norms with recommended feeding practices (Mukuria et al. 2016; Nguyen et al. 2019; Scott et al. 2018). Successful interventions employed various engagement strategies, such as structured dialogue sessions and community mobilisation that recognised grandmothers as agents of change within their households (Mondal et al. 2023; Saggurti et al. 2018). In contrast, local health authorities in India took a different approach, embedding peer facilitators directly within the government system to build institutional support (Komal et al. 2014; Singh et al. 2017; Singh et al. 2017; Vir et al. 2014). Nonetheless, the legitimacy of these efforts remained challenged by entrenched, layered cultural and institutional norms, primarily from family elders who influenced support uptake, the shortage of change agents and institutional coverage barriers (Newton‐Lewis and Bahety 2021; Warren et al. 2020; Yorick et al. 2021). These barriers were evident in both cultural practices that conflicted with recommended feeding behaviours and systemic constraints that limited programme reach and sustainability (Mondal et al. 2023; Saggurti et al. 2018).

Misinformation and scepticism from household gatekeepers eroded trust in nonprofessional support. This misinformation resulted from competing social and child‐feeding norms that remained dominant in traditional communities in LMICs, thereby reducing uptake and continuity of participation (Jahir et al. 2021; Mukuria et al. 2016; Nguyen et al. 2019; Scott et al. 2018; Singh et al. 2017). As a result, contradictory information undermined trained peer support and challenged their credibility, particularly when professional endorsement was absent (Arifeen et al. 2009; Jahir et al. 2021; Singh et al. 2017). Studies have also shown that patriarchal norms, women's domestic responsibilities and time constraints reduced enrollment (Jahir et al. 2021; Mukuria et al. 2016; Nguyen et al. 2019; Saville et al. 2018; Scott et al. 2018).

#### 3.3.3 Implementation and Structural Inefficiencies

Operational barriers, including fragmented coordination and logistical issues such as long distances and delays in government approvals, hindered peer support activities, such as regular meetings. These distance and transportation challenges contributed to low attendance by community health workers at training sessions (Jahir et al. 2021; Yorick et al. 2021). Further, inadequate communication materials, including poor quality visual aids, lack of locally adapted messages, and insufficient training and tools, compromised programme engagement (Jahir et al. 2021; Kim et al. 2015; Sanghvi et al. 2024). These constraints were compounded by perceived inequity in resource distribution for peer support activities, which has led to distrust within peer support systems, declining community acceptance and refusal to attend sessions (Jahir et al. 2021; Scott et al. 2018).

Additionally, weak follow‐up mechanisms often fail to capture and act on the early reductions in engagement, such as infrequent home visits. These mechanisms did not identify underperformance early or ensure accountability, which gradually weakened peer support. For example, supervisees, even when checked, rarely received advice on how to improve their performance or overcome constraints, which prevented continuous improvement (Kim et al. 2015). These engagement and follow‐up gaps contributed to volunteer attrition, which, combined with a weak supervisory system, threatened programme effectiveness and reach (Kim et al. 2015; Newton‐Lewis and Bahety 2021). In turn, such inconsistent oversight led to deviations from guidelines, compromising implementation fidelity and quality (Kim et al. 2015; Mukuria et al. 2016; Yorick et al. 2021).

## 4 Discussion

This theory‐informed systematic review of 32 studies identified key factors influencing peer support for complementary feeding practices in LMICs. Community ownership was facilitated by engagement of local leaders and families, while equitable access was achieved through inclusive incentives and mentorship, which enhanced enrollment (Jahir et al. 2021; Mondal et al. 2023; Mukuria et al. 2016; Nguyen et al. 2019; Rahman et al. 2023; Saggurti et al. 2018; Scott et al. 2018; Singh et al. 2017; Singh et al. 2017; Warren et al. 2020; Yorick et al. 2021). Interventions built trust and credibility by recruiting credible women and aligning with existing social structures and norms, thus enhancing the legitimacy of peer support (Kim et al. 2015; Menon et al. 2016; Newton‐Lewis and Bahety 2021; Nguyen et al. 2019). Sustained peer support programmes required integration into existing systems (activation), with ongoing support provided through refresher courses, supervisory oversight, and balanced incentive packages (Kim et al. 2019; Rahman et al. 2023; Singh et al. 2017; Vir et al. 2014; Yorick et al. 2021). Studies that integrated peer networks into health systems (institutionalisation) and used multimodal communication strategies improved complementary feeding practices (Flax et al. 2021; Kim et al. 2019; Menon et al. 2016; Mukuria et al. 2016; Newton‐Lewis and Bahety 2021; Rawat et al. 2017; Sanghvi et al. 2016; Saville et al. 2018; Shi et al. 2010; Suresh et al. 2019; Yorick et al. 2021; Younes et al. 2015). These strengths should be leveraged.

Certain facilitators and barriers shift in their influence on peer support over time. During the early intervention phase, fathers and older women emerged as drivers of scepticism. Yet, involving them in the interventions could reverse these dynamics. Donor‐driven transition cycles and programme adaptation during scaling also created resource mismatches with expanded peer support networks. These mismatches contributed to a decline in supervision quality. This decline led to demotivation and attrition, especially as the programme underwent strategic changes after the withdrawal of the funding partner. This implies that the relationship between the subconstructs is not static.

Community engagement and ownership of peer support in LMICs show promise for improving complementary feeding by enhancing enrollment and acceptance of these interventions. Community engagement encourages people to work together to address cultural norms and traditions that hinder the adoption of recommended feeding practices, including gender roles and decision‐making processes (Ayu et al. 2024). Peer support workers are often local individuals who serve as models for adopting innovations early or are experienced individuals trusted as sources of information and support. Women in the peer network are already familiar with one another, and the interventions made the network more organised while embedding evidence‐based and culturally tailored messages. Peer support interventions often utilise the existing familiar social environment (Muslihah et al. 2022; Rahman et al. 2023), which can be leveraged in the co‐design of digital tools and media content to increase the exposure and intensity of support (Flax et al. 2022).

Peer support interventions are dependent on external funding, which remains the case even when evidence shows the urgent need to leverage a strong delivery mechanism that can work at scale (Bhutta et al. 2008). Yet, it has also been recognised that the magnitude of malnutrition cannot be addressed by donor‐driven and government programmes alone (Raymond et al. 2018). This raises the question of where to strike a balance by developing a funding model. In this context, the Sustainable Development Goals (SDG 17.14) promote partnerships for goals and emphasise integrating sustainability considerations across interventions and policies (United Nations General Assembly 2015). Despite these aspirations, our review revealed that interventions had practical limitations due to ongoing resource constraints and sustainability issues in the post‐study period. These weaknesses in planning have become a threat to sustainability indicators. Consequently, refresher courses, mentorship, follow‐up, and incentives have been gradually phased out, which has also weakened the cohesion of peer support groups. Altogether, these findings demonstrated major drawbacks in the intervention design framework. To address these issues, a harmonised peer support system with sustainable funding models, responsible exit strategies, and a post‐intervention plan is needed (Glenn et al. 2021).

Most reviewed studies were funded by donor organisations, such as USAID and various philanthropic entities, funding approaches that proved to be unsustainable. Recent experience with shifts in US policy resulted in the abrupt withdrawal of support, which adversely impacted critical services and triggered crises and political upheaval (Zumla et al. 2025). This donor dependency highlights the urgent need for a political commitment to overcome the systemic barriers to the financial sustainability of peer support, particularly when such commitment translates into operational action (Fox et al. 2015; Fracassi et al. 2020).

To ensure effective peer support for complementary feeding initiatives, policymakers should prioritise sustainable funding and establish a clear agenda. This agenda should guarantee ongoing support for multisectoral and community‐based approaches. Although political commitment often lacks the requisite financial resources (Fox et al. 2015; Fracassi et al. 2020), strong leadership can help solve issues of poor governance, accountability, and transparency. Such issues divert resources from local peer support systems. Over time, strong leadership can also build a sense of national ownership. Donors also must act ethically and predictably to back domestic efforts. They need to show genuine commitment to community ownership by aligning with existing systems, supporting integration, and outlining a clear plan for long‐term sustainability (Morley and Silver 2023).

Effective peer support interventions require streamlined volunteer recruitment, training, motivation, and retention. We found that peer support systems often weaken due to various household, community, and institutional challenges. These weaknesses persist because existing policies and commitments lack cohesive laws, strategies, and clear implementation plans. As a result, key challenges such as poor resourcing, fragmented efforts, and uneven access to peer support remain unaddressed (Torlesse et al. 2022). Targeted advocacy strategies, such as highlighting the social and economic costs of malnutrition, have catalysed change by raising nutrition higher on the agenda in Africa (African Union Commission et al. 2014). A similar approach could reveal the costs, both monetary and non‐monetary, of not investing in community‐led platforms, such as peer support systems, to make complementary feeding support accessible and reduce inequality. This advocacy can provide grounds for enforcing legislation that supports peer networks as a viable, sustainable platform for early years nutrition, including complementary feeding practices (Harris et al. 2017).

Our stakeholder analysis revealed inconsistencies and limited involvement of households and key community informants. This limited involvement has been documented in a previous mixed‐methods review (Martin et al. 2020). That review found only one peer‐led complementary feeding study with practical family involvement, and another scoping review also showed a limited number of studies (Martin et al. 2021). There has been inadequate representation of different sectors, such as agriculture and education. This reflects a lack of coordination and gaps in the policy landscape that necessitate multisectoral collaboration. This has led sectors to work in isolation, creating operational hardships that could have been addressed if there were common platforms for collaboration and knowledge sharing (Gaihre et al. 2019). A weakened peer support system has negative effects that extend beyond early‐year nutrition, as it serves as a platform for other essential health promotion activities. This aligns with the Essential Nutrition Actions, which call for multisectoral action beyond the health system and emphasise people‐centred action (World Health Organization 2019). Without strong collaboration to support the peer network system, fragmented and redundant efforts can erode trust in future interventions. Therefore, partners must consider efforts to strengthen the peer support system, as it is the most accessible support option for mothers. Its failures extend beyond nutrition promotion and the project lifespan. Strengthening the system requires accountability and investment in building a trusting, multisectoral partnership.

Involving key stakeholders in designing and running peer support programmes can help build stronger collaborations that pool resources more effectively, identify practical challenges early and enable individuals to take ownership of and address solutions (Ramakrishnan and Webb Girard 2020). Operational research is needed to understand strategies that mitigate and prevent the gradual weakening of once‐effective peer support initiatives in India, Bangladesh, and Vietnam. Such research can reveal the specific contribution of each sector in ensuring that the peer network remains active and that mothers receive consistent and reliable support (Torlesse et al. 2022).

The lack of continuous support for peer support workers leads to limitations in epistemic legitimacy, as peer supporters are perceived as lacking the necessary expertise to warrant confidence in their recommendations (Nguyen et al. 2019; Warren et al. 2020). This will threaten future peer‐based interventions, as investment in oversight, feedback, and skill support, which drives participation momentum, is often short‐lived. In low‐income settings where peer support workers are not economically better off, incentives drive their recruitment and commitment. The effectiveness of peer support workers often depends on their perceived credibility and trustworthiness within the community (Ramakrishnan and Webb Girard 2020). Therefore, the legitimacy of peer support seems central to its effectiveness, as it depends on community approval and performance, which fosters confidence in their skill sets. Continuous monitoring facilitates ongoing problem identification and continuous quality improvement with peer support. This enables the support system to meet existing and emerging public health nutrition needs.

The strengths of this systematic review include its pragmatic, theory‐informed approach, which offers valuable insights into engagement processes in peer support systems. It framed a complex, multilayered actor dynamics influencing peer support through the lens of cognitive participation in the NPT. Additionally, the systematic review encompassed diverse cultural and health system contexts. The detailed analysis of barriers and facilitators, including when and how they operate, provides implementers, policymakers, and researchers with practical suggestions for designing interventions and adjusting policies. Despite these strengths, the review has limitations. First, the subconstructs of cognitive participation may be insufficient to capture the intricate dynamics of peer support revealed through quantitative assessment. While qualitative findings were included, these insights were constrained by the limited availability of formative research and evaluative studies, which often lack sufficient detail and depth.

## 5 Conclusion

Financial, structural, sociocultural, and capacity‐related factors contributed to the implementation of peer‐led complementary feeding support. While high levels of commitment during the early phases of intervention facilitated implementation, a gradual loss of momentum transformed these facilitators into barriers to progress. In particular, limitations in peer support undermined its legitimacy, which ultimately led to attrition and the dissolution of the peer group. To counter this, a strong monitoring mechanism can identify and mitigate these factors as they shift from facilitators to barriers. These practical considerations are key to sustaining the peer support network and integrating it into the broader healthcare system.

## Author Contributions

A.I., R.L., and K.C. contributed to conceptualising and developing the methodologies for this systematic review. A.I. and H.S. undertook the literature search, screening and data extraction. A.I. and H.S. drafted the manuscript, and all authors were involved in drafting and critically revising it. They also approved the final version for publication.

## Funding

The authors received no specific funding for this work.

## Conflicts of Interest

The authors declare no conflict of interest.

## Supporting information

supmat.

## Data Availability

Data sharing not applicable to this article, as no datasets were generated or analysed during the current study.
