# Supplementary material for: Barriers and Facilitators to Cognitive Participation in Peer Support for Complementary Feeding in LMICs: A Theory‐Informed Systematic Review
Source: Matern Child Nutr. 2026 Jan 8;22(1):e70154. doi: 10.1111/mcn.70154 (PMC12780870; doi:10.1111/mcn.70154)
Supplement: Supplementary file 1 — supmat. [file MCN-22-e70154-s001.docx]

**Supplementary file 1. Search Strategy for PICO**

| **Keywords** |  |
| --- | --- |
| Low and Middle Income Countries | “low and middle income countr*” or “low middle income countr*” or LMIC or Afghan* or Albania* or Algeria* or Angola* or Argentina* or Armenia* or Azerbaijan* or Bangladesh* or Belarus* or Beliz* or Benin* or Bhutan* or Bolivia* or Bosnia* or Herzegovin* or Botswan* or Brazil* or Bulgaria* or Burkina* or Burundi* or “Cabo Verde*” or “Cape Verde*” or Cambodia* or Cameroon* or “Central African” or Chad* or China or Chinese or Colombia* or Comor* or Congo* or “Costa Rica*” or “Ivory Coast” or Cuba* or Djibouti* or Dominica* or Ecuador* or Egypt* or “El Salvador*” or Eritrea* or Ethiopia* or Filipino* or Fiji* or Gabon* or Gambia* or Georgia* or Ghana* or Grenad* or Guatemala* or Guinea* or Guyan* or Haiti* or Hondura* or Hungar* or India* or Indonesia* or Iran* or Iraq* or Jamaica* or Jordan* or Kazakhstan* or Kenya* or Khmer or Kiribati* or Korea* or Kosov* or Kyrgyz* or Lao* or Leban* or Lesotho* or Liberia* or Libya* or Macedonia* or Madagascar* or Malawi* or Malaysia* or Maldiv* or Mali* or “Marshall Island*” or Mauritania* or Mauriti* or Mexic* or Micronesia* or Moldova* or Mongolia* or Montenegr* or Morocc* or Mozambi* or Myanma* or Burmese or Namibia* or Nauru* or Nepal* or Nicaragua* or Niger* or Nigeria* or Pakistan* or Palau* or Panama* or “Papua New Guinea*” or Paraguay* or Peru* or Philippines or Filipino or Romania* or Russia* or Rwanda* or Samoa* or “Sao Tome*” or Senegal* or Serbia* or Seychell* or “Sierra Leon*” or “Solomon Island*” or Somalia* or “South Africa*” or Sudan* or “Sri Lanka*” or “St Lucia*” or “Saint Lucia*” or “Saint Vincent” or “St Vincent” or Grenadines or Surinam* or Swazi* or Syria* or Tajikistan* or Tanzania* or Thai* or Timor* or Togo* or Tonga* or Tunisia* or Turk* or Turkmenistan* or Tuvalu* or Uganda* or Ukrain* or Uzbekistan* or Vanuatu* or Venezuela* or Vietnam* or “West Bank” or Gaza or Yemen* or Zambia* or Zimbabwe* |
| Complementary feeding practices | (complementary feeding or introduction of solids or infant feeding or weaning or bottle feeding or formula feeding or formula-fed or prelacteal feeding or infant feeding or breastfeeding or complementary feeding or, formula feeding or, responsive feeding or minimum dietary diversity or minimum meal frequency or minimum acceptable diet or supplementary feeding) OR (MM "Infant Nutritional Physiology+") OR (MM "Infant Feeding, Supplemental") OR (MM "Feeding of Persons with Disabilities") OR (MM "Infant Feeding Schedules") OR (MM "Infant Feeding+") OR "complementary feeding" OR (MM "Bottle Feeding") OR (MM "Infant Feeding Pattern Impairment (Saba CCC)") OR (MM "Ineffective Infant Feeding Pattern (NANDA)") OR (MM "Feeding Methods+") OR (MM "Eating Behavior+") OR (MM "Bottle Feeding (Iowa NIC)") |
| Peer support | AB (peer support or peer counseling or peer mentor or peer mentors or peer mentoring or peer group or support group or community health worker or lay health advisor or lay health workers or lay person or expert by experience or peers or mother-to-mother support groups or self-help groups or support groups or women groups or Community Health Workers or Community Networks or peer network) OR (promotores OR promotora OR promotoras OR embajadoras OR comodrones OR abuela OR "lay advocates" OR "lay health" OR "lay workers" OR "lay worker" OR "lay advisors" OR "lay educators" OR "lay counselors" OR "lay counselor" OR "indigenous volunteers" OR "lay health workers" OR "lay health worker" OR "lay facilitators" OR "community health advisor" OR "community health advisors" OR "community health workers" OR "community health worker" OR "peer workers" OR "peer educators" OR "peer educator" OR "natural helpers" OR consejeras OR "community health volunteers" OR "community health volunteer" OR "community educators" OR "community educator" OR "village health workers" OR "village health worker" OR "community based distributors" OR community health aides[MeSH]) |
| Study design | AB (mixed methods or mixed method or mixed-method or mixed methods or 'qualitative' and 'quantitative' or participatory design or codesign or codesign or co design or participatory action research or community-based participatory research or participatory or pragmatic intervention or community participation or community engagement or community involvement or formative assessment or formative feedback or formative evaluation) |

Supplementary file 2. Quality criteria checklist: Primary research

| **Authors** | **Overall quality rating**  **Negative (-), Neutral (**∅**), Positive (+)** | **Comments** |
| --- | --- | --- |
| **(Acharya et al., 2019)** | **+** | Lack of blinding  Limited withdrawal reporting |
| **(Arifeen et al., 2009)** | **+** | Lack of blinding  Potential for residual confounding |
| **(Das et al., 2016)** | **+** | Lack of blinding  Potential ecological fallacy |
| **(Flax, Fagbemi, et al., 2022)** | **∅** | The lack of blinding introduced bias in self-reported outcomes. Staff from the funding organisation were involved in the design of the implementation; intervention spillover limits the interpretability of group comparisons. |
| **(Flax et al., 2021)** | **∅** | Spillover effects  Potential biases due to lack of blinding and reliance on self-reported data  Data collection challenges due to external factors (e.g., civil unrest, COVID-19).  Residual confounding |
| **(Gope et al., 2019)** | **+** | The nonrandomized design  Self-reported data  Lack of blinding  Limited details are available on attrition or loss to follow-up across time points. |
| **(Jahir et al., 2021)** | **∅** | Potential interviewer and recall bias due to self-reported data  Lack of blinding  lack of a control group  potential for social desirability bias |
| **(Kim et al., 2015)** | **∅** | Provided valuable insights into implementation fidelity  Absence of a control group  Short implementation period  Inconsistent supervision practices  Inconsistent tool dissemination |
| **(Kim et al., 2018)** | **+** | The sustainability of SBCC interventions in improving IYCF practices  Reduced program intensity after donor support |
| **(Kim et al., 2019)** | **+** | Intervention exposure variability across regions  Lack of blinding  Potential spillover effects |
| **(Komal et al., 2014)** | **∅** | The nonrandomized design  The absence of a control group  Lack of blinding  Limited details are available on attrition or loss to follow-up across time points. |
| **(Menon et al., 2016)** | **∅** | Lack of blinding; outcomes based on maternal recall, not observed behaviours; and program funders involved in design. |
| **(Mondal et al., 2023)** | **+** | The non-randomised pre-post design is employed, and it is well managed and transparently discussed. |
| **(Mukuria et al., 2016)** | **+** | Potential selection bias, but efforts were made to select comparable communities.  Some baseline differences in marital status and education between groups |
| **(Newton-Lewis & Bahety, 2021)** | **∅** | The lack of significant improvement in feeding practices  Inconsistent intervention coverage  Lack of blinding |
| **(Nguyen et al., 2019)** | **+** | Self-reported data  Lack of blinding |
| **(Olney et al., 2015)** | **+** | Lack of blinding  Short program duration and seasonal influence |
| **(Rahman et al., 2023)** | **+** | Limitations in blinding due to the cross-sectional design are acknowledged and discussed.  Lack of blinding  Funded by an external organisation |
| **(Rawat et al., 2017)** | **+** | Lack of blinding  Self-reported data |
| **(Roche et al., 2017)** | **∅** | The nonrandomized design, self-reported feeding practices and no reported power calculation. |
| **(Saggurti et al., 2018)** | **+** | Lack of blinding  Potential bias due to self-reported outcomes |
| **(Sanghvi et al., 2016)** | **+** | Lack of blinding  Reliance on self-reported data |
| **(Saville et al., 2018)** | **+** | Lack of blinding  Reliance on self-reported |
| **(Scott et al., 2018)** | **+** | Lack of blinding  Potential biases from self-reported and retrospective data  A sequential study (quantitative followed by qualitative) may not fully capture changes over time. |
| **(Shi et al., 2010)** | **+** | Lack of blinding  Limited details on the handling of missing data and participant attrition.  Reliance on self-reported data |
| **(V. Singh et al., 2017)** | **∅** | Lack of blinding and reliance on self-reported data  Residual confounding due to baseline differences between groups |
| **(A. Singh et al., 2017)** | **∅** | Lack of blinding  Self-reported data  Short follow-up period (12 months)  Lack of detailed reporting on participant withdrawals and missing data |
| **(Suresh et al., 2019)** | **+** | Lack of blinding and lack of information on potenitial confounders, like NGOs. |
| **(Vir et al., 2014)** | **∅** | Lack of randomisation and blinding  Reliance on self-reported data introduces potential biases  The absence of baseline data limits pre- and postintervention comparisons  Limited discussion on handling missing data and attrition |
| **(Warren et al., 2020)** | **+** | Lack of detailed reporting on participant withdrawals.  Potential bias due to the nonblinded design. |
| **(Yorick et al., 2021)** | **∅** | potential selection bias  lack of blinding  Incomplete data on withdrawal and attrition  Small sample sizes |
| **(Younes et al., 2015)** | **∅** | Self-reported data  Potential selection bias |
